# Dr. Scott, on White Vitriol in Ague

**Published:** 1804-02-01

**Authors:** Walter Scott

**Affiliations:** Stamfordham, Northumberland


### *To the Editors of the Medical and Physical Journal.*

GENTLEMEN,

FROM the liberality which I have experienced from you, and from no other cause, I have presumed to offer the following remarks on Mr. Cuming's communication, on the use of white vitriol in agues.

Specifics are so fashionable, that the profession of a physician is nearly at an end, for almost every disease has its specific; and where that is supposed to be wanting, electricity, galvanism, &c. &c. or that great reflection upon common sense, the metallic tractors, are seriously recommended; O tempora, O mores!

I have had occasion to make use of white vitriol some years ago in the cure of ague. I must say I have a good opinion of it; I think it answered the intention pretty well, but I thought cuprum ammoniacum* rather preferable as a tonic in the cure of ague.

The solutio mineralis arsenici, has often disappointed me in the cure of ague, which first led me to make use of those medicines.

I never much admired this solution, I always supposed (not to enter fully upon the subject) that it injured the tone of the stomach, and from consent, the brain, nervous system, &c.

I have very frequently given those tonics with very great success in the cure of hooping cough, and periodical pain of the head, &c. but I will not say they deserve the name of specifics.

---

* Cupr. ammon. ℈j. Mic. panis ʒij. Syr. cort. aurant. q. s. m. f. pill. No. xxiv. Capt. j. vel ij.—iij. (sensim augendo dosin) hora decubitus quotidie.

Cinchona has with me frequently failed in the cure of ague; I have often been much astonished how this medicine acquired so very high a character; I am confident it does not deserve it.

Dr. James's powder, so much celebrated in the cure of fever, has not, in my opinion, done more harm than cinchona.

The word specific, I very much dislike; to me the sound is empirical; it is wonderful that I should not be acquainted with one; even in lues, in some particular cases under certain circumstances, I have seen mercury fail of the desired effect.

My patients could never take the quantity of white vitriol* mentioned by Mr. Cuming; in my practice, and from observation, I cannot think it deserves the epithet specific.

I certainly agree with Mr. Cuming that it is a medicine worthy the attention of medical gentlemen, and this is all I would wish to say; for if the practitioners in Essex and Kent, hope to meet with a specific in white vitriol in the cure of ague, they will be miserably disappointed. I believe a sea voyage, or a long journey, will answer in those cases as a more powerful specific. I have always thought so; and indeed, to be candid, which I sincerely wish to be, I must say, that during the time the Northumberland Militia was quartered in Essex, that I never could cure the hardy Northumbrian, so long as the remote cause was constantly applied. The inhabitants resist the disease in a certain degree, perhaps from habit; and in them, the cure is less difficult.

*Stamfordham, Northumberland,* I am, &c.

*December* 19, 1803. WALTER SCOTT, M.D.

P.S. Zincum. The preparations of zinc are employed principally in external applications, as ophthalmics. Internally they have been recommended in epilepsy and other spasmodic affections, both alone, and with cuprum ammoniacum; and some think they prove an *useful* addition to the Peruvian bark in intermittents. Vid. Edinburgh Dispensatory, p. 265.

---

* Zinc. calcinat. gr, vj. Chel. cancr. ppt. ʒiß. Sacch. alb. pulv. ʒß. m. f. pulv. in chartul. vj. dividend. capt. j. mane et vesperi quotidie.

